# Dysregulated immunometabolism in gut inflammation

**DOI:** 10.3724/abbs.2025192

**Published:** 2025-10-21

**Authors:** Mengqi Zheng, Qiuheng Tian, Jing Shen, Shiyang Li

**Affiliations:** 1 Department of Gastroenterology Qilu Hospital of Shandong University; Advanced Medical Research Institute Shandong University Jinan 250012 China; 2 Shandong Provincial Clinical Research Center for Digestive diseases Jinan 250012 China; 3 Key Laboratory for Experimental Teratology of Ministry of Education Shandong University Jinan 250012 China

**Keywords:** immunometabolism, intestinal disorder, inflammation

## Abstract

Gut inflammatory diseases, including inflammatory bowel disease (IBD), infectious enteritis, and other inflammatory conditions, are among the most common non-neoplastic intestinal disorders. Their pathogenesis is often driven by an imbalance between pro-inflammatory and anti-inflammatory signals, with immune cells playing pivotal roles in maintaining this equilibrium. Immune cells in the gut exhibit complex, multifaceted functions: they eliminate pathogens, promote tissue repair, and counteract tumors, but excessive immune activation can exacerbate tissue damage and disease progression. Notably, metabolic reprogramming in inflammatory contexts serves as a key regulator of immune cell function and phenotypic switching. This includes alterations in cellular energy metabolism ( *e*. *g*., macrophage polarization via disrupted glycolysis or fatty acid oxidation) and the modulation of immune responses by microenvironmental metabolites ( *e*. *g*., bile acid-mediated Th17/Treg balance). While alterations in immune cell function and composition within the inflammatory milieu are well-established, the significance of disease-associated metabolic reprogramming—specifically how metabolism regulates immune cell function—has garnered increasing attention. This review explores how cellular metabolic reprogramming, changes in the metabolic microenvironment, and gut dysbiosis collectively influence the differentiation, proliferation, and function of immune cells in various intestinal inflammatory diseases, as well as their impact on disease progression.

## Introduction

Intestinal homeostasis is crucial for human health, enabling efficient nutrient absorption, protecting against pathogen invasion, and maintaining immune balance. Disruption of this equilibrium leads to various inflammatory disorders of the gut, including inflammatory bowel disease (IBD), a chronic, non-specific inflammatory condition encompassing ulcerative colitis (UC) and Crohn’s disease (CD), and infectious enteritis caused by pathogenic bacteria, fungi, viruses, or parasites, as well as other inflammatory diseases, such as necrotizing enterocolitis (NEC). If left unchecked, these conditions cause progressive tissue damage, increase the risk of systemic infection, and, in severe cases, lead to sepsis, multi-organ failure, or malignant transformation [1].

The immune system is central to maintaining intestinal homeostasis, providing crucial protection under normal conditions. However, when immune responses become dysregulated, either through excessive activation or loss of tolerance, they can drive intestinal inflammation. In IBD, aberrant immune responses, such as inappropriate activation of macrophages, dysregulated T helper (Th) cells, and impaired regulatory T cells (Tregs), along with compromised epithelial barrier integrity, lead to the uncontrolled secretion of pro-inflammatory cytokines and sustained mucosal injury [1]. In infectious enteritis, the immune system’s ability to promptly recognize pathogens via pattern recognition receptors is critical. Effective innate immune responses, including neutrophil recruitment and antimicrobial peptide release, along with the activation of pathogen-specific adaptive immunity, are essential for pathogen clearance [2]. Failure in these mechanisms leads to persistent infections, extensive tissue damage, and potential systemic dissemination. In other inflammatory diseases, such as NEC and Celiac disease, immune cells also act as primary responders to tissue injury, as their activation triggers inflammatory cascades that influence both disease severity and recovery [ 3, 4] . Ultimately, the delicate balance between protective immunity and pathological immune responses determines the susceptibility, clinical manifestations, and disease course of gut inflammation.

Metabolism in immune cells is not merely a source of energy but a dynamic and integral regulator of immune activation, function, and fate, particularly in the context of gut inflammation [ 5, 6] . This concept, known as metabolic reprogramming, plays a critical role in both the pathogenesis and resolution of gut inflammation. In the inflamed intestinal microenvironment, characterized by hypoxia, fluctuating nutrient availability, and microbial stimuli, immune cells undergo profound metabolic shifts to meet increased functional demands [7]. For example, glycolysis is upregulated to support effector functions, such as neutrophil antimicrobial activity and macrophage-driven pro-inflammatory cytokine production [8]. Oxidative phosphorylation (OXPHOS) and fatty acid oxidation (FAO) are preferentially utilized by long-lived memory cells and Tregs, supporting their survival and suppressive capacity [9]. Crucially, these metabolic transitions actively shape immune cell behavior. Metabolic intermediates and cofactors, such as succinate [10], itaconate [11], acetyl-CoA [12], and NAD⁺ [6], serve as powerful signaling molecules that regulate key inflammatory pathways ( *e*. *g*., HIF-1α [13], mTOR [14], and AMPK [15]), influence transcription factor activity ( *e*. *g*., Foxp3 stability in Tregs [10]), drive epigenetic modifications, and maintain redox balance. As a result, cellular metabolism directly impacts immune cell differentiation, activation thresholds, cytokine profiles ( *e*. *g*., IL-1β, TNF-α, and IL-10), migratory capacity, and survival [16]. When immunometabolism becomes dysregulated, immune cell dysfunction occurs across different forms of gut inflammation.

This review explores the emerging field of immunometabolism in gut inflammation, focusing on how metabolic reprogramming in key immune populations, such as macrophages, neutrophils, innate lymphoid cells (ILCs), and T cells, affects the progression and resolution of intestinal inflammation. We delve into how alterations in key metabolic pathways, including glycolysis, OXPHOS, FAO, and amino acid metabolism, influence immune cell function within the context of intestinal inflammatory diseases. By understanding the intricate interplay between metabolism and immune response, this review seeks to illuminate potential therapeutic strategies that target immunometabolic pathways, with the aim of restoring immune homeostasis and reducing chronic inflammation.

## Macrophages

### Macrophage immunometabolism

**Figure 1:**
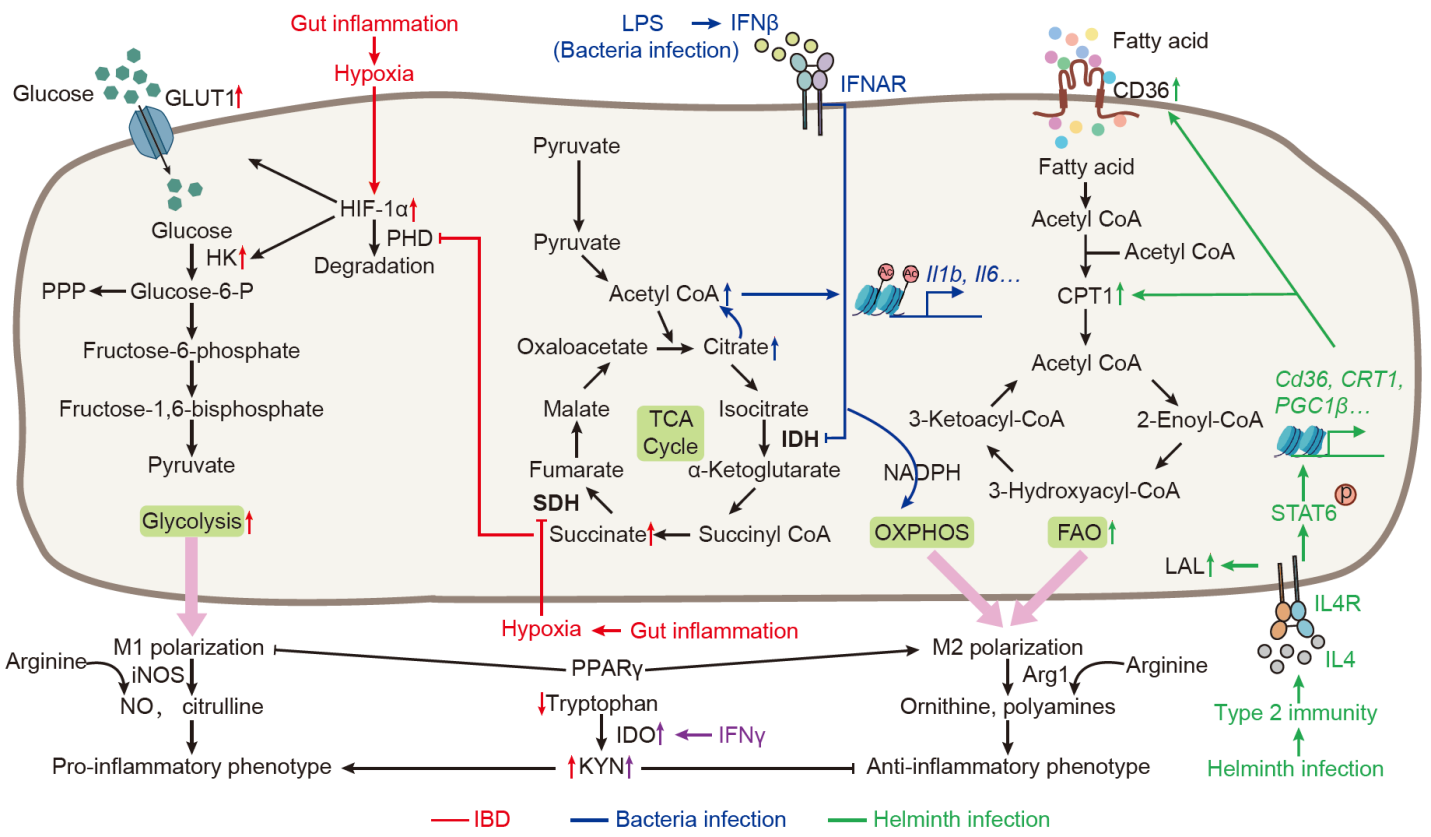
Figure 1 Metabolic regulation of macrophages during gut inflammatory diseases Macrophage polarization is critically modulated by distinct metabolic pathways: glycolysis promotes the pro-inflammatory M1 phenotype, whereas oxidative phosphorylation (OXPHOS) and fatty acid oxidation (FAO) support the anti-inflammatory M2 phenotype. Under hypoxic conditions induced by local inflammation, hypoxia-inducible factor 1-alpha (HIF-1α) expression is upregulated, enhancing glucose uptake through glucose transporters (e. g., GLUT1) and glycolysis via key enzymes such as hexokinase (HK). Hypoxia also leads to succinate accumulation, which inhibits prolyl hydroxylase (PHD) activity, thereby stabilizing HIF-1α and reinforcing M1 polarization. During bacterial infection, lipopolysaccharide (LPS) -induced IFNβ signaling suppresses the tricarboxylic acid (TCA) cycle, resulting in acetyl-CoA accumulation. This promotes histone acetylation at proinflammatory cytokine gene loci (e. g., Il1b, Il6), augmenting their expression. In contrast, IL-4/STAT6 signaling during parasitic infection upregulates FAO-related genes (e. g., CD36, CPT1), driving M2 polarization and anti-parasitic responses. PPARγ-mediated fatty acid signaling further enhances M2 polarization while suppressing M1 programs. Arginine metabolism delineates a classical metabolic dichotomy: inducible nitric oxide synthase (iNOS)-mediated metabolism promotes proinflammatory responses, while Arg1 activity supports anti-inflammatory phenotypes. Reduced tryptophan levels in inflammatory environments, coupled with IFNγ-induced indoleamine 2,3-dioxygenase (IDO) overexpression, enhance kynurenine (KYN) production, facilitating M1 polarization.

Intestinal macrophages are vital components of the intestinal immune system, widely distributed in the intestinal mucosal lamina propria. They play key roles in maintaining intestinal homeostasis and regulating the pathological processes of IBD. Under normal conditions, macrophages help preserve intestinal stability by phagocytosing pathogens, maintaining barrier integrity, and interacting with the gut microbiota [5]. However, during gut inflammation, intestinal macrophages undergo significant metabolic reprogramming that drives their functional polarization and profoundly influences disease progression and therapeutic responses. These context-dependent metabolic states are critical not only for initiating effective antimicrobial responses but also for regulating inflammation and promoting tissue repair ( Figure 1).

The metabolic state of macrophages tightly couples with their functional phenotype—the pro-inflammatory M1 phenotype primarily relies on glycolysis for energy supply, while the anti-inflammatory M2 phenotype depends on OXPHOS and FAO [5]. Consequently, metabolic remodeling of glucose and lipids is a key feature of macrophage function in intestinal inflammatory diseases, especially for IBD and infectious enteritis. It has been shown that hypoxia-inducible factor-1 alpha (HIF-1α) promotes the expression of glycolysis-related genes such as glucose transporter 1 (GLUT1) and hexokinase 2 (HK2), thereby enhancing glycolysis and M1 polarization [17], while hypoxia is the most prevalent condition observed in the intestinal inflammatory microenvironment. Meanwhile, elevated serum and fecal levels of succinate were found in IBD patients due to impaired succinate dehydrogenase (SDH) activity during hypoxia, which further inhibits prolyl hydroxylase (PHD) that promotes HIF-1α stabilization, creating a pro-inflammatory feedback loop that further inhibits mitochondrial function [ 10, 18] . Similarly, excessive succinate accumulation is also observed in bacterial infectious enteritis, as LPS-induced tricarboxylic acid (TCA) cycle impairment leads to citrate accumulation and subsequent SDH inhibition, thereby reinforcing glycolytic flux and pro-IL-1β synthesis-a hallmark of the inflammatory Warburg effect [ 19– 21] . Furthermore, accumulated citrate is shunted into parallel pathways: cytosolic ATP-citrate lyase converts it to acetyl-CoA, fueling histone acetylation for inflammatory gene activation ( *e*. *g*., *Il1b*, and *Il6*). Conversely, metabolic processes associated with M2 macrophage polarization, such as OXPHOS, are significantly suppressed in macrophages from IBD patients [22], accompanied by mitochondrial dysfunction and increased oxidative stress. These factors collectively induce metabolic reprogramming in macrophages, predominantly featuring a shift from OXPHOS to glycolysis. This transition thereby promotes M1 polarization and pro-inflammatory phenotype in macrophages under inflammatory conditions, especially the acute phase of IBD.

In contrast, the chronic IBD presents distinct pathophysiological features. Although M1 polarization persists, cytokines such as IL-4, IL-13, and IL-10 induce metabolic reprogramming in macrophages, favoring polarization toward the M2 phenotype (especially the M2a subtype), and thereby supporting tissue repair [23]. Notably, M2 macrophages contribute to tissue regeneration by secreting TGF-β and upregulating arginase activity. TGF-β activates fibroblasts and stimulates collagen production [24], while arginine metabolism supports epithelial proliferation, exerts immunosuppressive functions via polyamine generation, and provides proline for collagen synthesis [25]. However, excessive activation of M2 macrophages, particularly the pro-fibrotic M2a and M2c subtypes induced by IL-4/IL-13 and IL-10, respectively, leads to the secretion of pro-fibrotic mediators such as TGF-β and platelet-derived growth factor [ 26, 27] . These factors activate myofibroblasts, enhancing their proliferation and the synthesis of extracellular matrix components, particularly collagen types I and III, thereby promoting fibrotic remodeling. This process can result in intestinal stricture, a common complication of chronic IBD [28]. Targeting macrophage metabolic reprogramming, through inhibition of mitochondrial function, reduction in oxidative phosphorylation and glycolysis, and impairment of cholesterol efflux, may promote M2b polarization over the pro-fibrotic M2a/M2c subtypes, potentially preventing tissue fibrosis in chronic IBD [29].

However, unlike in IBD, M2 macrophage polarization is common in helminth infections and other parasitic challenges, as it is primarily driven by type 2 cytokines such as IL-4 and IL-13, which are robustly produced in this context. The IL-4/13-STAT6 signaling cascade promotes metabolic reprogramming toward FAO, thereby fueling mitochondrial OXPHOS—a bioenergetic foundation essential for tissue repair and anti-inflammatory functions [30]. IL-4-induced STAT6 activation transcriptionally upregulates key components of the FAO machinery, including CD36 (for fatty acid uptake), CPT1 (for mitochondrial fatty acid transport), and PGC1β (for mitochondrial biogenesis), collectively sustaining OXPHOS and supporting M2 macrophage effector functions [31]. Traditionally, fatty acids for FAO are supplied via classical lipolysis, in which adipose triglyceride lipase mobilizes triacylglycerols stored in lipid droplets. However, evidence highlights a non-canonical pathway essential for M2 activation: lysosomal lipolysis [32]. Notably, IL-4 has been shown to upregulate lysosomal acid lipase, which hydrolyzes endocytosed lipid cargoes into free fatty acids, thereby directly providing substrates for mitochondrial FAO [32].

In addition to shifting energy supply modes, metabolic reprogramming also alters cellular nutrient availability, which exerts profound implications on macrophage polarization and function. Lipid metabolism exhibits pleiotropic roles in regulating macrophage function. Choline, an essential nutrient and precursor for membrane phospholipids, is metabolically required for M2 polarization and optimal immunity against intestinal helminth infections [33]. While the short-chain fatty acid butyrate promotes macrophage antimicrobial specialization by inhibiting histone deacetylase 3 (HDAC3), thereby orchestrating a metabolic program involving suppression of both glycolysis and mTOR signaling—without compromising inflammatory cytokine production [34]. However, the immunoregulatory role of M2 macrophages is a double-edged sword. In certain infectious contexts, M2 polarization may facilitate pathogen persistence as PPARα-mediated FAO facilitates M2 polarization and has been shown to exacerbate *Salmonella* *typhimurium* infection [35]. In contrast, impaired PPARγ and FAO activity caused by NF-κB activation during IBD has been shown to be closely linked to intracellular lipid accumulation and the sustained pro-inflammatory phenotype in macrophages [36].

Amino acid metabolism also plays a crucial role in macrophage reprogramming during gut inflammation. Arginine metabolism diverges in M1 and M2 macrophages: M1 macrophages metabolize arginine via the inducible nitric oxide synthase (iNOS) pathway to produce nitric oxide (NO) and citrulline, which drives inflammation [37]. In contrast, M2 macrophages utilize the Arg1 pathway to produce ornithine and polyamines, which play protective roles in IBD, including promoting colonic epithelial regeneration and facilitating Treg differentiation [37]. In IBD, insufficient arginine supply drives macrophage polarization toward M1 and induces the iNOS pathway of arginine metabolism, while arginine supplementation alleviates colitis [38]. In IBD glutamine—an important energy donor—also shows significant reduction [39]. Under these conditions, macrophage metabolic pathways shift: reduced α-ketoglutarate causes succinate accumulation, which inhibits PHD and stabilizes HIF-1α, thereby driving macrophages toward M1 polarization [40]. Additionally, glutamine deficiency promotes M1 polarization by reducing mTORC1 signaling pathway activity and increasing cellular oxidative stress levels [41], thereby exacerbating the progression of colitis. Intriguingly, worms can evade immune clearance by reprogramming macrophage metabolism via transcellular delivery. The helminth *Heligmosomoides polygyrus* subverts macrophage function through its glutamate dehydrogenase (GDH), which is internalized via the macrophage Fcγ receptor CD64. GDH enzymatic activity converts glutamine to α-KG and oncometabolite 2-hydroxyglutarate, concomitantly suppressing leukotriene C4 synthase to block pro-inflammatory leukotriene synthesis. This dual rewiring of macrophage epigenetics and metabolism amplifies PGE2-mediated Th2 suppression, thereby dampening anti-helminth immunity [42]. Tryptophan metabolism in macrophages primarily occurs towards the kynurenine (KYN) pathway, and KYN and its metabolite serotonin regulate inflammatory responses. In UC, tryptophan concentration decreases while kynurenine pathway activity increases, leading to elevated KYN production and suppression of macrophage anti-inflammatory functions [43]. Inflammatory cytokines such as IFN-γ upregulate indoleamine 2,3-dioxygenase (IDO1) expression, further disrupting macrophage metabolism and exacerbating inflammation [44].

Metabolic regulation in macrophages also plays a critical role in some other types of gut inflammatory diseases. In NEC, intestinal macrophage infiltration is significant elevated [45]. Activation of macrophage α7nAChR reduces NLRP3 inflammasome activation by modulating mTOR phosphorylation, which, through the mTORC1/HIF-1α signaling pathway, plays a key role in regulating macrophage glycolysis, thereby alleviating intestinal inflammation and injury in NEC [14]. Additionally, FK866-mediated depletion of intracellular NAD ^+^ restrains macrophage infiltration and M1 polarization, thereby alleviating symptoms in experimental NEC pups [46]. Inflammatory injury is often occurred after radiation, during which macrophages accumulate in the intestinal stem cell regions, promoting the reprogramming and proliferation of epithelial cells to a fetal-like state, and macrophage deficiency impairs this process [47]. Ascorbic acid, a potent antioxidant, promotes the restoration of the M2 response, as well as tissue remodeling and wound healing, thereby mitigating radiotherapy-induced intestinal damage [48]. In Celiac disease, gliadin peptides trigger higher levels of IL-8 and TNF-α production by macrophages [49]. This pro-inflammatory cytokine secretion is accompanied by higher levels of expression of M1 markers, like CD80, CD86, and CD40, and increased activation of the NF-κB signaling [50]. In contrast, gliadin is also observed to trigger M2-like shift of macrophages [51]. In an experimental murine Celiac disease model, rapamycin–gliadin composite nanoparticles drive macrophage metabolic reprogramming from glycolysis toward OXPHOS. This metabolic shift, concomitant with elevated serum itaconate levels, potentiates inter-organ immunoregulatory crosstalk—expanding splenic PD-L1⁺ tolerogenic DCs while suppressing pathogenic Th1 cells—ultimately promoting gluten tolerance and alleviating intestinal inflammation [52].

### Potential therapeutic strategies targeting macrophage immunometabolism

Changing the metabolic process in macrophage is commonly achieved via suppressing pro-inflammatory M1 polarization or promoting anti-inflammatory M2 polarization. For example, inhibition of glycolysis by 2-deoxy-D-glucose (2-DG) targeting HK or PFK15 targeting key glycolytic enzyme PFKFB3 attenuates M1 polarization [ 53, 54] . Alternatively, supplementation with itaconate restores mitochondrial function and promotes M2 polarization, offering a therapeutic avenue for NEC in newborns [11].

In this review, we highlighted the regulatory role of the glycolysis-OXPHOS/FAO metabolic switch in macrophage polarization. Therapeutics targeting this switch can directly influence macrophage fate and their associated pro- or anti-inflammatory functions. For instance, dioscin, a steroidal saponin, not only suppresses M1 polarization via the mTORC1-HIF-1α pathway but also promotes M2 polarization through mTORC2/PPARγ signaling [55]. This dual modulation rebalances macrophage metabolism from M1- to M2-favoring state, thereby exerting anti-inflammatory and tissue-repair effects that significantly ameliorate UC. Similarly, polysaccharide of *Radix Paeoniae Alba* facilitates a metabolic shift from glycolysis to OXPHOS in macrophages, repolarizing them toward a restorative phenotype and offering therapeutic potential in IBD [56]. Furthermore, restoring microenvironmental homeostasis is essential to recalibrate metabolic balance in cases where polarization dysregulation is driven by alterations in the tissue microenvironment. Fecal microbiota transplantation, for example, can reconstruct the gut microbiota and correct microbiota-derived metabolic abnormalities, such as imbalanced BAs, thereby reversing macrophage polarization defects, suppressing intestinal inflammation, and promoting tissue repair [ 57, 58] . These metabolic interventions suppress acute inflammation and mitigate fibrosis in chronic colitis by precisely regulating macrophage differentiation [29]. Thus, reprogramming cellular metabolism or rectifying aberrant metabolic reprogramming in inflammatory settings represents a critical avenue for macrophage-targeted therapy in intestinal inflammatory diseases.

## DCs

### DC immunometabolism

DCs are central to regulating immune responses in the intestinal mucosa, balancing immune tolerance and activation through antigen recognition and T cell differentiation. Like macrophages, DCs’ metabolic states are tightly linked to their immune functions: quiescent DCs rely on OXPHOS for energy homeostasis, while activated DCs shift to glycolysis to fuel inflammatory responses [59]. Activated DCs also increase GLUT1 expression to facilitate IL-12/IL-23 secretion [60].

Tolerogenic DCs (tolDCs), essential for Treg differentiation, are functionally supported by catabolism-centered metabolism, characterized by enhanced glycolysis in combination with elevated OXPHOS and FAO [61]. Tryptophan metabolism is a core regulatory mechanism in tolDCs and IDO-high tolDCs suppress effector T cells through the modulation of co-stimulatory molecules, namely CD86 downregulation and PD-L1 upregulation [62]. In IBD, IDO activity is reduced in tolDCs, shifting the local T cell environment toward a pro-inflammatory state [63]. Bile acid-activated FXR signaling suppresses inflammatory responses by limiting DC activation and promoting Treg differentiation, thus controlling excessive inflammation [ 64, 65] .

Unlike homeostatic conditions, when pathogens invade the lamina propria during intestinal infection, the tolerogenic response triggered by CD103 ^+^ DCs must be switched to a proinflammatory response to protect the host [66]. Following bacterial infection, human intestinal DCs enhance the production of proinflammatory cytokines such as TNF, IL-1β, and IL-23, a process that depends on glycolytic reprogramming [66].

### Potential therapeutic strategies targeting DC immunometabolism

The harnessing of tolDCs represents a promising therapeutic strategy for the treatment of inflammatory disease. AMPK serves as a central regulator of cellular metabolism, whose activation inhibits fatty acid synthesis while enhancing FAO. Pharmacological activation of AMPK in human monocyte-derived DCs induces a tolerogenic phenotype, evidenced by an enhanced capacity to prime functional Tregs [67]. This process drives metabolic reprogramming, characterized by increased glycerophospholipid degradation, mitochondrial fission-induced FAO, and enhanced glucose catabolism [68]. The conversion of tolDCs is dependent on catabolic metabolism, which is further reinforced through the induction of autophagy and downregulation of protein synthesis via inhibition of mTORC1. Thus, rapamycin could inhibit DC maturation and effector functions, thereby preserving the tolerogenic properties of DCs [69] to suppress intestinal inflammation. In addition, IDO is an effective immunosuppressive enzyme in DC, as acetylated STAT-3, CTLA-4, and PD-1, have been shown to maintain tolDCs activity through IDO [70]. Upregulating IDO expression helps maintain tolDCs, making it a potential therapeutic target for treating gut inflammation.

## ILCs

**Figure 2:**
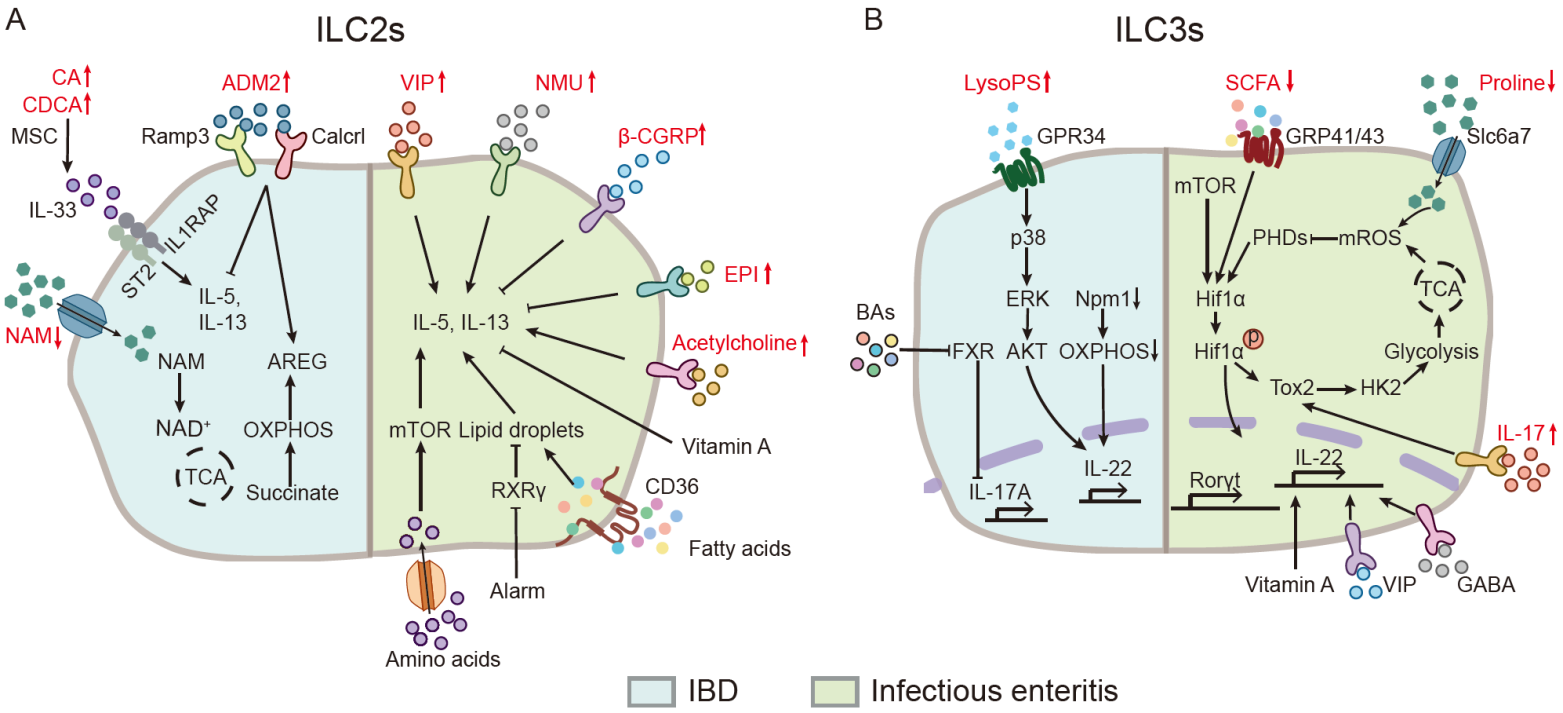
Figure 2 Metabolic regulation of ILCs during gut inflammatory diseases (A) Disrupted gut metabolic pathways shape ILC2 function and modulate IBD pathology. Elevated primary bile acids, cholic acid (CA) and chenodeoxycholic acid (CDCA), skew intestinal ILC2s toward a pro-inflammatory phenotype. Reduced intestinal NAD⁺ levels in ulcerative colitis and liver cirrhosis impair ILC2 survival and attenuate amphiregulin (AREG)-mediated tissue repair. In contrast, increased adrenomedullin 2 (ADM2) expression promotes AREG-dependent tissue-protective responses. During anti-parasitic infection, enhanced lipid metabolism and neuropeptide signaling fine-tune ILC2 function. Increased fatty acid uptake supports ILC2 responses, while neuropeptides such as vasoactive intestinal peptide (VIP), neuromedin U (NMU), and acetylcholine promote ILC2 activity. In contrast, beta-calcitonin gene-related peptide (β-CGRP) and epinephrine (EPI) suppress ILC2 function. Additionally, amino acids are essential for supporting type 2 immunity, whereas vitamin A deficiency promotes type 2 inflammatory response. (B) Metabolic perturbations influence intestinal immunity and IBD pathogenesis through the modulation of ILC3 function. Dysregulated OXPHOS impairs IL-22 production, while disrupted bile acid metabolism inhibits farnesoid X receptor (FXR) and promotes the expression of pro-inflammatory IL-17A, exacerbating IBD. In contrast, lysophosphatidylserine (LysoPS) released by neutrophils enhances IL-22 secretion, protecting the intestine from inflammatory damage. During infection-driven colitis, reduced levels of SCFAs and proline destabilize HIF1α, impairing the antibacterial activity of ILC3s. Conversely, the neuropeptides gama-aminobutyric acid (GABA) and VIP promote IL-22 production and enhance antimicrobial activity. Vitamin A is essential for IL-22-dependent antibacterial defense.

ILCs, which include NK cells, ILC1s, ILC2s, ILC3s and lymphoid tissue inducer (LTi) cells, are critical modulators of mucosal immunity, inflammation, and tissue homeostasis [71]. Alterations in the intestinal inflammatory environment and metabolic composition impact ILCs function, thereby influencing the progression of colitis ( Figure 2).

### ILC1 immunometabolism

NK cells and non-cytotoxic ILC1s, drive inflammation and facilitate pathogen clearance through the secretion of pro-inflammatory cytokines such as IFN-γ and TNF-α [72]. Increased ILC1 activity and numbers correlate strongly with the onset and progression of IBD. Studies have shown significantly elevated ILC1 numbers in the intestinal tissues and peripheral circulation of IBD patients, with ILC1 levels correlating with disease severity in CD [ 73, 74] . ILC1s dominate as IFN-γ producers during acute *Toxoplasma gondii* [75] and *Clostridioides difficile* infection [76]. Despite their well-established role as rapid responders, the metabolic landscape of ILC1s in gut inflammation remains poorly understood.

### ILC2 immunometabolism

ILC2s play a dual, context-dependent role in intestinal inflammation: while they drive type 2 inflammatory responses during allergy and parasitic infection, they also promote tissue repair, particularly during the resolution phase. Their metabolic programs are highly plastic and dynamically tuned by activation state. Naïve human blood ILC2s, which rely predominantly on OXPHOS, switch to glycolysis and mTOR signaling upon IL-33 activation to sustain cytokine production, while maintaining OXPHOS through amino acid metabolism to support proliferation and cellular fitness [77]. This functional versatility is further shaped by microenvironmental inputs—including neural signals, metabolites, and cytokines. The dysregulation of intestinal ILC2s, whether by altered abundance or impaired function, can drive intestinal inflammation [ 74, 78] .

In the inflamed intestine, metabolic disturbances profoundly alter ILC2 activity and thereby aggravate disease progression. In IBD, microbiota-derived bile acids such as cholic acid (CA) skew intestinal ILC2s toward a pro-inflammatory phenotype, characterized by loss of AREG and elevated IL-5 expression, exacerbating colitis and tissue damage [79]. Likewise, reduced intestinal NAD ^+^ in UC and liver cirrhosis, compromise intestinal ILC2 survival and AREG-mediated tissue repair through decreased succinate production and impaired OXPHOS, ultimately delaying the resolution of colitis [6].

During helminth challenge, activated ILC2s integrate glycolysis and FAO to fuel IL-13 production [80]. In parallel, alarmin-mediated suppression of retinoid X receptor-γ impairs cholesterol efflux, promoting neutral lipid accumulation and thereby enhancing type 2 inflammation in the small intestine [81].

Neuro-immune crosstalk provides additional context-dependent regulation of ILC2 function. Neuron-derived vasoactive intestinal peptide (VIP) and neuromedin U (NMU) potentiate ILC2-mediated protection against helminths [ 82, 83] . ILC2-derived acetylcholine promotes autocrine population expansion of ILC2s, ensuring optimal anti-helminth type 2 immunity [84]. In contrast, this activation is counter-regulated by inhibitory signals: ILC2-derived IL-13 feedbacks onto intrinsic enteric neurons to induce β-CGRP release, which suppresses ILC2 responses and anti-helminth immunity [85]. Similarly, epinephrine (EPI) signaling via the β _2_-adrenergic receptor (β _2_AR) potently repress ILC2 responses [86]. The context-dependent role of neuro-immune crosstalk is further highlighted by adrenomedullin 2 (ADM2), a CGRP-related peptide from enteric neurons that promotes the AREG-dependent tissue-protective functions of ILC2s during DSS-induced colitis [87].

Nutrient availability and metabolite utilization further dictate ILC2 function and disease outcome. Amino acid uptake controls the magnitude of ILC2 responses in part via tuning of mTOR, with loss of the transporters SLC7A5 and SLC7A8 diminishing type 2 immunity during helminth infection [ 88, 89] . Vitamin A deficiency further amplifies ILC2 responses by expanding populations and enhancing IL-13 overproduction [90]. Collectively, these findings underscore how ILC2 metabolic plasticity integrates environmental, microbial, neuronal, and nutritional cues to balance inflammation and tissue repair in the intestine.

### ILC3 immunometabolism

ILC3s are pivotal regulators of mucosal immunity, mounting rapid IL-17, IL-22 and GM-CSF responses against extracellular bacteria and fungi. Their functional state is tightly coupled to metabolic reprogramming, with a switch from OXPHOS to glycolysis upon activation to support effector responses [91]. This transition is coordinated by mTORC1, which not only drives glycolytic metabolism but also stabilizes HIF1α and sustains RORγt expression, thereby supporting proliferation and IL-22/IL-17 production [92]. Mitochondrial ROS further reinforce this metabolic shift by stabilizing HIF1α, integrating redox signals with metabolic control to enhance ILC3 activity [92]. ILC3 plasticity extends beyond acute immune responses. Following infection with *Citrobacter rodentium*, a subset of ILC3s acquires a long-lived “trained” phenotype, marked by a metabolic switch from glycolysis and glutaminolysis to enhance TCA cycle activity, OXPHOS, and fatty acid synthesis, which enhances memory-like function [92]. Thus, ILC3s rely on metabolic pathways to not only mediate immediate effector functions but also establish memory responses.

Dysfunction or imbalance in ILC3s, particularly a reduction in IL-22-producing subsets, is a hallmark of intestinal inflammation [92]. These defects are closely tied to metabolic dysregulation within the inflamed gut. Disruption of OXPHOS in ILC3s during colitis further impairs IL-22 production and tissue regeneration [93]. In NEC, diminished short-chain fatty acid (SCFA) availability promotes FXR-driven ferroptosis of neonatal epithelial cells, releasing lipid peroxides that blunt IL-22 secretion by ILC3s [94]. During colitis-associated epithelial injury, apoptotic neutrophils release lysophosphatidylserine (LysoPS), which engages GPR34 on ILC3s to initiate tissue repair programs [95].

Amino acid metabolism provides critical checkpoints for ILC3 activity. For example, proline, which is captured by LTi cells via SLC6A7, acts as a metabolic hub, generating mitochondrial ROS to sustain activation and cytokine production. Loss of SLC6A7 or dietary proline restriction impairs LTi cells responses and worsens colitis in mice [96]. Moreover, reduced circulating proline in UC patients may contribute to disease progression [97], suggesting proline supplementation could be a therapeutic strategy. By activating the aryl hydrocarbon receptor (AhR), tryptophan metabolites orchestrate host protection against *Citrobacter rodentium* infection by suppressing ILC2 function and enhancing ILC3 maintenance [98].

Microbial metabolites also exert broad control. SCFAs enhance IL-22 secretion via GPR41/43 and HDAC inhibition [ 99, 100] , and acetate coordinates neutrophil-ILC3 crosstalk against *Clostridium difficile* infection via FFAR2 [101]. Perturbations of this axis, such as hyperbaric oxygen-induced SCFA depletion, impair HIF-1α-IL-22 signaling and exacerbate infection severity [102]. Bile acids, regulated by FXR, are also critical to ILC3 function. In IBD, diminished intestinal FXR signaling promotes ILC3-dependent intestinal inflammation by IL-17A production [102]. Bile acid metabolic dysregulation in IBD patients [103] could cause ILC3 dysfunction and exacerbates colitis. Of note, Vitamin A deficiency depletes ILC3s, impairing IL-22-dependent antibacterial defense [90].

In conclusion, ILC3s serve as metabolically sensitive sentinels, integrating dietary, microbial, and neural cues to balance mucosal immunity, barrier protection, and inflammation. Dysregulation of these pathways in gut inflammation not only contributes to disease pathogenesis but also opens avenues for therapeutic interventions targeting metabolic circuits.

### Potential therapeutic strategies targeting ILC immunometabolism

Fatty acids are essential for the expansion of ILC2 during helminth infection. Treating mice with a pan retinoic acid receptor signaling inhibitor increased fatty acid acquisition in ILC2s, promoting their accumulation and enhancing their protective function [80]. Additionally, lovastatin, a competitive inhibitor of 3-hydroxy-3-methylglutaryl coenzyme A (HMG-CoA) reductase, the rate-limiting enzyme in cholesterol biosynthesis, reduces lipid droplet accumulation in ILC2s [81]. This reduction in lipid accumulation may help attenuate ILC2s pro-inflammatory activity and ameliorate intestinal inflammation.

A high-fiber diet can protect the gut by promoting the expansion of ILC2 and ILC3 through the production of SCFAs [104]. However, the dietary fiber inulin may increase the production of CA, which in turn promotes the pro-inflammatory activation of ILC2 [105]. Therefore, caution is warranted when using dietary fiber as a therapeutic intervention, as its impact on ILC function may vary depending on the type of fiber.

mTOR activation is crucial for the metabolic and functional activation of both ILC2s and ILC3s [92]. In contrast, mTOR inhibition, achieved through conditional mTORC1 ( *Rptor*) knockout or mTOR inhibitors such as rapamycin, dampens the activity of ILC2s and ILC3s [92]. The modulation of mTOR—whether by activation or inhibition, can influence ILC behavior, ultimately affecting the severity of intestinal inflammation. The balance between mTOR activation and inhibition in ILCs is a crucial factor in the pathogenesis and treatment of inflammatory disorders in the gut.

The stability of HIF-1α, driven by hypoxia, mTOR activation, mROS or SCFAs, positively regulates ILC3 function, thereby conferring protection against pathogenic infection and ameliorating colitis [102]. Pharmacological activation of HIF-1α, for example through PHD inhibition, represents a promising therapeutic strategy. Although inhibition of HIF-1α has been shown to attenuate allergic airway inflammation by restraining ILC2 metabolism and function [105], the precise mechanisms by which HIF-1α regulates intestinal ILC2s and its impact on intestinal inflammation remain poorly understood, highlighting an important direction for future investigation.

Mitochondrial oxidation of succinate by SDH is critical for maintaining the electron transport chain (ETC) and the production of mROS, which are essential for the proper function of ILC2s and ILC3s [ 6, 92] . Inhibition of SDH by dimethyl malonate disrupts the functionality of both ILC2s and ILC3s. Targeting SDH to modulate ILC2 and ILC3 function could offer a promising strategy to control intestinal inflammation.

## Neutrophils

### Neutrophil immunometabolism

Neutrophils, traditionally viewed as inflammatory effector cells in gut inflammation, are now recognized for their dual function in both tissue damage and repair [106]. This duality is intricately linked to the dynamic remodeling of their metabolic state. In a healthy intestine, neutrophils transiently infiltrate the mucosa to eliminate pathogens and secrete factors that promote tissue repair. However, in the context of chronic inflammation, hypoxic and high-lactate conditions induce persistent activation and metabolic reprogramming, leading to functional imbalance [107]. In this process, neutrophil metabolic programs rapidly shift from OXPHOS and FAO to glycolysis and the pentose phosphate pathway (PPP) to ensure rapid ATP generation while producing substantial NADPH to fuel oxidative burst [108]. The chronic inflammatory environment in IBD skews neutrophil metabolism toward a pro-inflammatory phenotype, establishing “metabolic memory” that exacerbates disease progression [109]. Chemotaxis and local activation are essential for neutrophil function in the intestine. In IBD, enhanced eicosanoid metabolism, particularly via leukotriene B4 (LTB4), may contribute to the recruitment of neutrophil to sites of inflammation [ 110, 111] . Furthermore, the reduction of SCFAs caused by gut microbiota dysbiosis in IBD patients contributes to neutrophil infiltration and the release of pro-inflammatory cytokines, as SCFAs can inhibit the production of neutrophil-derived pro-inflammatory factors, reduce their infiltration, and suppress neutrophil extracellular trap (NET) formation through HDAC inhibition [112]. These pathways collectively amplify neutrophil-associated local inflammation.

In infectious enteritis, neutrophils act as frontline responders that rapidly migrate to sites of intestinal infection, where they play a critical role in pathogen clearance. They eliminate invading microbes through multiple effector mechanisms, including phagocytosis, the generation of ROS, degranulation, and the release of NETs. Effective neutrophil recruitment to the intestine is essential for preventing infectious colitis [ 110, 113] . Their functional plasticity during intestinal infection is underpinned by metabolic reprogramming within tissue-specific microenvironments. A key metabolic shift occurs during neutrophil maturation: while immature neutrophils rely on FAO-driven mitochondrial respiration, mature neutrophils predominantly utilize glycolysis [8]. This metabolic adaptation enhances host defense, for instance, GPR120-mediated upregulation of glycolysis in neutrophils boosts ROS and NET production, thereby increasing their bactericidal activity against *Citrobacter rodentium* [114]. Similarly, ROS generation via DUOX2 acts as a metabolic checkpoint in colonic neutrophils, with DUOX2 deficiency impairing the containment of *Salmonella typhimurium*, underscoring the importance of oxidative metabolism in pathogen control [109]. In *Clostridioides difficile* infection, tryptophan catabolism in cecal lamina propria cells mitigates immunopathology by limiting the accumulation of IFN-γ-producing neutrophils [115].

### Potential therapeutic strategies targeting neutrophil immunometabolism

Current research on neutrophil-targeted therapies for gut inflammatory diseases primarily focuses on inhibiting neutrophil recruitment, exemplified by agents such as Danirixin, which targets CXCR2, and Efalizumab, directed against CD11a [ 116, 117] . This strategy capitalizes on the characteristic trafficking of neutrophils to inflammatory sites during active disease phases. As previously discussed, neutrophil maturation and activation are associated with a metabolic shift toward heightened glycolysis, which is necessary to meet the elevated energy demands for effector functions. Consequently, interventions targeting glucose uptake or critical enzymatic steps within the glycolytic pathway can markedly attenuate the pro-inflammatory activity of neutrophils, as evidenced by reduced generation of ROS and neutrophil NETs [ 118, 119] .

Moreover, neutrophil-derived ROS play dual roles depending on the context: they are essential for antimicrobial defense during infections, yet contribute significantly to tissue damage and inflammation in IBD. Since ROS production depends on activation of the PPP to supply NADPH, modulating this pathway offers a means to contextually regulate neutrophil oxidative burst. For instance, CYR5099, an inhibitor of NADPH oxidase 2 (NOX2)—a key enzyme responsible for respiratory burst and ROS generation—has been shown to effectively suppress neutrophil ROS production [120]. Additionally, restoration of the gut microbiota or direct supplementation with SCFAs inhibits HDAC, thereby reducing neutrophil recruitment and pro-inflammatory cytokine secretion [112].

It is important to note, however, that these metabolic pathways are conserved across multiple cell types. Therefore, achieving cell-specific targeting is essential for minimizing off-target effects. For example, encapsulating metabolic inhibitors in nanoliposomes functionalized with neutrophil-specific antibodies such as anti-Ly6G may enable precise delivery to neutrophils, enhancing therapeutic specificity [118].

## T Cells

### Th17 and Treg immunometabolism

**Figure 3:**
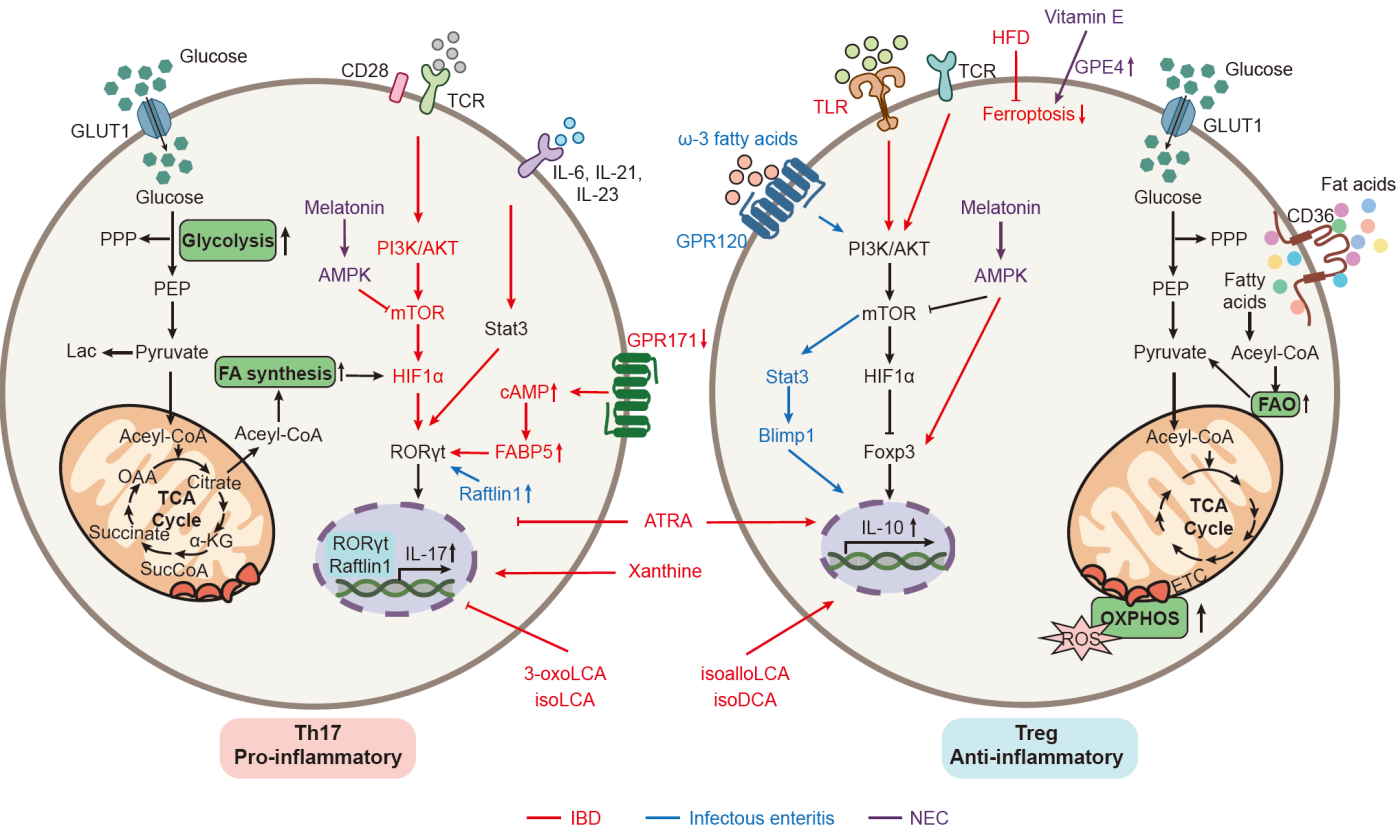
Figure 3 Metabolic regulation of T cells during gut inflammatory diseases T cell differentiation is critically regulated by distinct metabolic pathways: glycolysis promotes the differentiation of T cells into pro-inflammatory Th17 cells, whereas OXPHOS and FAO support the proliferation and differentiation of Treg cells. In IBD, activation of the PI3K/AKT/mTOR signaling pathway upregulates HIF-1α expression, which enhances the expression of the Th17 master transcription factor RORγt while suppressing the expression of the Treg key transcription factor Foxp3. Loss of GPR171 results in elevated cAMP levels, leading to CREB phosphorylation and enhanced FABP5 expression, thereby driving excessive Th17 activation and aggravating colitis. A high-fat diet induces ferroptosis of intestinal Treg cells, thereby disrupting immune tolerance and initiating the development of colitis. In infectious colitis, Raftlin1 organizes cholesterol/sphingolipid-enriched lipid rafts that promote RORγt activation, thereby enhancing Th17 pathogenicity. Conversely, the ω-3 fatty acid receptor GPR120 promotes IL-10 secretion by Treg cells through the PI3K/AKT/mTOR pathway. In NEC, vitamin E enhances GPX4 expression, thereby inhibiting Treg ferroptosis and subsequently alleviating intestinal damage and inflammation. Melatonin ameliorates NEC by restoring the intestinal Th17/Treg balance, inhibiting the differentiation of pathogenic Th17 cells, and enhancing the generation of protective Treg cells through the activation of the AMPK/SIRT1 pathway. In IBD, gut microbiota dysbiosis leads to abnormal bile acid metabolism, with a marked reduction in secondary bile acids and their derivatives. 3-oxolithocholic acid (3-oxoLCA) and isolithocholic acid (isoLCA) inhibit Th17 differentiation by suppressing RORγt activity, whereas isoallolithocholic acid (isoalloLCA) and isodeoxycholic acid (isoDCA) promote Treg differentiation via Foxp3. In addition, intestinal epithelial cells generate xanthine through the unfolded protein response, which promotes Th17 differentiation during colitis.

Th17 and Treg cells are two critical intestinal T cell subsets often discussed together due to opposing yet related developmental pathways. During gut inflammation, T cells undergo substantial metabolic reprogramming, which promotes their differentiation into distinct subtypes and supports diverse functional roles ( Figure 3). At steady state, Th17 cells utilize OXPHOS to maintain a relatively quiescent state. Upon activation, T cells undergo remarkable metabolic changes characterized by metabolic reprogramming and increased glycolysis to support biosynthesis and cellular functions [121]. As a short-lived inflammatory T cell subset, Th17 cells differentiation and function are primarily dependent on glycolysis rather than other metabolic pathways [122]. Inhibition of glycolysis, for example by treatment with 2-DG, can suppress Th17 cell development and cytokine production [123]. Additionally, in CD, OXPHOS seems to be also important for the function maintenance of Th17, as inhibiting OXPHOS *in vivo* directly weakens Th17 function and alleviates colitis [124]. The energy requirements and metabolic processes of Tregs are markedly different from those of Th17 cells. Compared with any other pathway, Treg cells rely more on OXPHOS and FAO as fuel sources for ATP generation and energy production [125]. Treg cells universally generate higher ROS concentrations than other T cells [126]. In IBD, TLR signaling activation enhances glycolysis and GLUT1 expression via the PI3K-Akt-mTORC1 pathway, promoting Treg proliferation. However, this quantitative Treg change occurs at the cost of reduced immunosuppressive capacity and Foxp3 expression [127]. Th17 cells play a critical role in host defense during infectious colitis, particularly at mucosal barriers such as the intestinal epithelium. They mediate protection against extracellular pathogens, including certain bacteria and fungi, through the production of pro-inflammatory cytokines like IL-17 and IL-22. However, their activity must be tightly regulated, as excessive or dysregulated Th17 responses can contribute to tissue damage and inflammation. This metabolic dichotomy is further shaped by the local gut microenvironment: for instance, homeostatic Th17 cells induced by commensal segmented filamentous bacteria utilize OXPHOS, maintaining a relatively quiescent state. In contrast, during pathogen-driven inflammation such as *Citrobacter rodentium* infection, Th17 cells shift to a Warburg-like glycolytic metabolism to support rapid proliferation and cytokine production [128]. AMPK is a heterotrimeric kinase complex that is involved in glucose uptake and glycolysis in various cell types [129]. AMPK deficiency leads to increased mTOR activity, accompanied by upregulated glycolysis and enhanced production of effector cytokines [130]. In contrast, AMPK is known to be particularly active in Treg cells. Activation of AMPK appears to drive the differentiation of naïve T cells into Treg cells by enhancing FAO, rather than into Th17 cells [131]. The development of NEC is associated with an increase in pro-inflammatory Th17 cells and a reduction in anti-inflammatory Tregs, resulting in T cell imbalance in the premature small intestine of both mice and humans. Melatonin ameliorates NEC by restoring the intestinal Th17/Treg balance, inhibiting the differentiation of pathogenic Th17 cells, and enhancing the generation of protective Treg cells through the activation of the AMPK/SIRT1 pathway [15].

In addition, other metabolic pathways are also involved in Th17 cell differentiation. Th17 cell differentiation and function highly depend on *de novo* fatty acid synthesis (FAS), with low reliance on exogenous fatty acids [12]. Acetyl-CoA carboxylase 1 (ACC1) and FASN—key FAS enzymes—activate transcription factor RORγt and promote IL-17 expression by generating long-chain fatty acids ( *e*. *g*., oleic acid, and stearic acid) [12]. Conversely, Treg differentiation primarily relies on FAO, mediated by carnitine palmitoyltransferase 1A (CPT1A) and significantly enhanced by short-chain fatty acids ( *e*. *g*., butyrate) [132]. In IBD, Th17 cells are excessively activated. GPR171 deficiency promotes Th17 cell differentiation through the cAMP-pCREB-FABP5 axis and induces lipid metabolic disorders, leading to intestinal inflammation in a FABP5-dependent manner [133]. A high-fat diet induces ferroptosis of intestinal Treg cells, thereby disrupting immune tolerance and initiating the development of colitis. Compared with effector T cells, Treg cells favor lipid metabolism and prefer polyunsaturated fatty acids (PUFAs) for the synthesis of membrane phospholipids. This inherent preference for polyunsaturated fatty acids triggers ferroptosis of Treg cells, which aggravates high-fat-diet-related colitis [134]. In infectious colitis, Raftlin1 scaffolds cholesterol/sphingolipid-enriched lipid rafts that facilitate RORγt activation, enhancing Th17 pathogenicity [135]. On the other hand, the ω-3 fatty acid receptor GPR120 promotes an OXPHOS-dependent anti-inflammatory program in IL-10–producing Treg cells, while its genetic loss exacerbates Th17-driven inflammation in infectious colitis models [136]. In NEC, vitamin E enhances GPX4 expression, thereby inhibiting Treg ferroptosis and subsequently alleviating intestinal damage and inflammation [137]. Moreover, microbiota dysbiosis-induced SCFA metabolic disturbances damage GPR43/GPR109A-mediated IL-10 secretion [138]. These factors collectively cause pro-/anti-inflammatory imbalance and amplify local inflammation.

Gut microbiota dysbiosis during IBD causes abnormal bile acid metabolism, with significant reductions in secondary bile acids and their derivatives. 3-oxolithocholic acid (3-oxoLCA) and isolithocholic acid (isoLCA) inhibit Th17 differentiation by suppressing RORγt activity, while isoallolithocholic acid (isoalloLCA) and isodeoxycholic acid (isoDCA) promote Treg differentiation via Foxp3 [139].

Several additional regulatory pathways fine-tune Th17 metabolism during gut inflammation. Glutathione synthesis via Gclc acts as a redox buffer, preserving IL-22 production by counteracting ROS-mediated mTOR hyperactivation during infection [140]. Moreover, xanthine derived from intestinal epithelial cells through unfolded protein response promotes Th17 differentiation during colitis [141]. In neonatal mice with experimental NEC, significant mucosal barrier damage was observed, accompanied by elevated pro-inflammatory cytokine levels and a reduction in Tregs [142]. Notably, retinoic acid, a metabolite of vitamin A, exerts protective effects by preventing T cell imbalance and reducing the severity of NEC in these mice [143].

### Th2 immunometabolism

T cells exert pivotal and dual functions in infectious colitis: mediating defensive immunity for pathogen clearance while maintaining mucosal barrier integrity. Critically, their functional specialization—balancing protective responses against dysregulated immunity—is governed by metabolic reprogramming and adaptations. Th2 immune responses to helminth infections play a critical role in host defense by preventing the survival of invading parasites during homologous secondary infections, expelling adult worms from the gut. HIF2α, which senses reduced oxygen availability, promotes Th2 cell differentiation in a cell-intrinsic manner and enhances CD4⁺ T cell-mediated immunity against gastrointestinal helminth infections [144]. During enteric infection with the helminth parasite *Heligmosomoides polygyrus*, Th2 cells in the small intestine exhibit elevated expression of several glycolytic enzymes compared to Th2 cells in mesenteric adipose tissue [145].

### CD8 ^+^ T cell immunometabolism

The current understanding of CD8 ^+^ T cells in IBD remains inconsistent, a disparity potentially attributable to their functional heterogeneity. Some subsets of tissue resident memory CD8 ^+^ T cells might be immunosuppressant, whereas others might exhibit pro-inflammatory features [146]. IBD frequently involves gut microbiota dysbiosis, leading to metabolic imbalance and metabolite abnormalities that directly impact CD8 ^+^ T cell behavior and cytokine secretion. Increased *Clostridium* proliferation in CD elevates secondary bile acids ( *e*. *g*., deoxycholic acid), activating the mTOR-OXPHOS axis in CD8 ^+^ T cells via TGR5 receptors and promoting excessive granzyme B and IFN-γ secretion, directly killing intestinal epithelial cells. TGR5 deletion in CD8 ^+^ T cells effectively alleviates ileitis, and portal vein secondary bile acid levels correlate positively with disease severity in ileitis patients [147]. Additionally, existing reports indicate potent regulatory effects of SCFAs on CD8 ^+^ T cells [148]; as the intestine is the primary site for SCFAs, dysregulated SCFA metabolism due to microbiota dysbiosis during IBD will severely impair CD8 ^+^ T cell function and homeostasis.

### Potential therapeutic strategies targeting T cell immunometabolism

Inhibiting the differentiation of pro-inflammatory Th17 cells or promoting the expansion of anti-inflammatory Tregs is potentially used to ameliorate gut inflammation. For instance, in IBD, CD226-deficient Tregs regulate the shift from OXPHOS to glycolysis through the AMPK/mTOR/Myc pathway. The use of 10058-F4 (a small-molecule c-Myc inhibitor) effectively restored the impaired function of CD226-deficient Tregs in an IBD model [149]. Additionally, proteomic analysis revealed that the drug-like ClpP agonist NCA029 chemically activates CD4 ^+^ T cells and inhibits OXPHOS and Th17 differentiation *in* *vitro*, alleviating symptoms of IBD [150]. In IBD, the novel BigLEN-Fc targets GPR171 to inhibit Th17 differentiation and significantly reduces inflammation in a mouse model [133].

However, unlike broad immunosuppressive approaches, a more refined strategy is to actively guide the Th17 to Treg phenotype conversion through metabolic reprogramming, based on the different metabolic needs of effector and regulatory immune cell populations. Such cell-specific modulation may reduce unnecessary side effects. For example, 2-DG, a classic glycolysis inhibitor, has been shown to inhibit Th17 development while promoting Treg induction [123]. Metformin, by activating AMPK and inhibiting mTORC1 signaling, reduces glycolysis rates and promotes the conversion of Th17 cells to Tregs, significantly alleviating experimental colitis in mice [151]. Furthermore, MTHFD2 inhibitors can induce a metabolic shift from glycolysis to OXPHOS, driving the conversion of Th17 cells to Treg-like cells and enhancing Foxp3 expression in a TGF-β-deficient microenvironment [152], improving experimental colitis models. Therefore, reprogramming the intrinsic metabolism of T cells has become a critical pathway for T cell-targeted therapies in intestinal inflammatory diseases.

## Conclusion and Future Perspectives

This review highlights how metabolic reprogramming directly shapes the phenotypes of several immune cell types, including macrophage, DCs, ILCs, neutrophils, and T cells, thereby influencing disease progression in IBD, infectious enteritis, NEC, and Celiac disease. However, more efforts are needed to understand whether metabolic alterations occur in other immune compartments, such as B cells, eosinophils, and basophils, and how these changes affect their function and clinical outcomes. It is also essential to elucidate the roles of specific metabolic enzymes and intermediates in immune regulation, as well as identify novel metabolic pathways and their contributions to cell-cell communications within the gut microenvironment. Of note, the spatiotemporal dynamics of gut microbiota and immune cell interactions will also provide critical insights into the phase-specific regulation of inflammation and tissue repair. Therefore, the integration of multi-omics technologies, particularly single-cell metabolomics, spatial transcriptomics, and metagenomics, could help resolve the spatial-temporal heterogeneous metabolic-immune-microbial cross-talk at high resolution. All these insights could unravel the complex regulatory networks driving gut inflammation, revealing novel targets for disease therapy.
